# Pentoxifylline Effects on Hospitalized COVID-19 Patients with Cytokine Storm Syndrome: A Randomized Clinical Trial

**DOI:** 10.3390/ph16040631

**Published:** 2023-04-21

**Authors:** Rania M. Sarhan, Ahmed E. Altyar, Ahmed Essam Abou Warda, Yasmine Mohamed Saied, Haytham Soliman Ghareeb Ibrahim, Mona F. Schaalan, Shaimaa Fathy, Neven Sarhan, Marian S. Boshra

**Affiliations:** 1 Clinical Pharmacy Department, Faculty of Pharmacy, Beni-Suef University, Beni-Suef 62511, Egypt; raniamohammad87@yahoo.com (R.M.S.); mariansobhy31@yahoo.com (M.S.B.); 2 Department of Pharmacy Practice, Faculty of Pharmacy, King Abdulaziz University, P.O. Box 80260, Jeddah 21589, Saudi Arabia; 3 Pharmacy Program, Batterjee Medical College, P.O. Box 6231, Jeddah 21442, Saudi Arabia; 4 Clinical Pharmacy Department, Faculty of Pharmacy, October 6 University, Giza 12585, Egypt; ahmedessamabouwarda@gmail.com; 5 Microbiology and Immunology Postgraduate Program, Faculty of Pharmacy, Cairo University, Cairo 11828, Egypt; yasmine.m.saied@gmail.com; 6 Cardiology Department, Faculty of Medicine, El-Fayoum University, El-Fayoum 63514, Egypt; drhaythamsoliman@gmail.com; 7 Clinical Pharmacy Department, Faculty of Pharmacy, Misr International University, Cairo 11828, Egypt; nevine.mohamed@miuegypt.edu.eg (M.F.S.); mona.schaalan@miuegypt.edu.eg (S.F.); shaimaa.fathy@miuegypt.edu.eg (N.S.)

**Keywords:** pentoxifylline, severe acute respiratory syndrome, cytokines release syndrome, COVID-19

## Abstract

COVID-19 is a fatal, fast-spreading pandemic, and numerous attempts are being made around the world to understand and manage the disease. COVID-19 patients may develop a cytokine-release syndrome, which causes serious respiratory diseases and, in many cases, death. The study examined the feasibility of employing legally available anti-inflammatory pentoxifylline (PTX), a low toxicity and cost medication, to mitigate the hyper-inflammation caused by COVID-19. Thirty adult patients who tested positive for SARS-CoV2 were hospitalized owing to the cytokine storm syndrome. They were given 400 mg of pentoxifylline orally TID according to the standard COVID-19 protocol of the Egyptian Ministry of Health. Besides this, a group of thirty-eight hospitalized COVID-19 patients who received the standard COVID-19 protocol was included in the study as a control group. The outcomes included laboratory test parameters, clinical improvements, and number of deaths in both groups. After receiving PTX, all patients showed a significant improvement in C reactive protein (CRP), and interleukin-6 (IL-6) levels at *p* < 0.01 and *p* = 0.004, respectively, while there was an increase in total leukocyte count (TLC) and neutrophil-to-leucocyte ratio (NLR) at *p* < 0.01 compared to their baseline levels. The D-dimer level showed a significant increase in the treatment group at *p* < 0.01, while showing no statistically significant difference in the control group. The median initial ALT (42 U/L) in the treatment group showed a decrease compared to the control group (51 U/L). No statistical significance was reported regarding clinical improvement, length of stay, and death percentages between the two groups. Our results showed no significant improvement of PTX over controls in clinical outcomes of hospitalized COVID-19 patients. Nevertheless, PTX displayed a positive effect on certain inflammatory biomarkers.

## 1. Introduction

COVID-19 is a virus-mediated disease that can cause a cytokine storm syndrome (CSS) in infected patients. Cytokine storm syndrome can be defined as a life-threatening clinical illness caused by a cascade of cytokine activation and characterized by overwhelming systemic inflammation, hyperferritinaemia, hemodynamic instability, and multiple organ failure (MOF) [1]. This pro-inflammatory response is essential to the damage that viral infections do to the patient and may be linked to worse clinical outcomes [2]. The vast majority of the adverse outcomes have been related, not to the cytotoxicity of the virus, but rather to an overt inflammatory reaction on the part of the patient. Moreover, a relationship has been established between a prolonged rise in pro-inflammatory cytokines and poor outcomes in ARDS patients [3].

Most likely, increased levels of several pro-inflammatory factors, including interleukin 1ß (IL-1ß), interferon (INF), monocyte chemoattractant protein 1 (MCP-1), IL-4, IL-7, IL-8, IL-9, and IL-10, as well as tumor necrosis factor (TNF-α), are the causes of the cytokine storm phase of the SARS-CoV-2 disease [4,5,6].

Several clinical interventions, including the IL-6-blocking drug tocilizumab, have been proposed to prevent or mitigate the severity of the cytokine storm [7]. There are no other therapeutic options that have been established up to this point for the purpose of mitigating the cytokine storm syndrome that is associated with COVID-19 in regular clinical research. Hence, phosphodiesterase (PDE-4) inhibitors such as theophylline and pentoxifylline are proposed as suitable COVID-19 therapeutic adjuvants [8,9,10].

Pentoxifylline possesses anti-inflammatory, anti-thrombotic, immunomodulatory, and antiviral effects, making it a viable alternative or supplemental therapy option for patients with moderate to severe symptoms [11]. Pentoxifylline is a methylxanthine derivative that inhibits phosphodiesterase 4 (PDE-4), which is a common and important regulator of cAMP metabolism in nearly all pro-inflammatory and immunological cells [12].

Pentoxifylline’s anti-inflammatory action is attributed to a decrease in the activation of pro-inflammatory cytokines such as TNF-α, interferon, and NF-B and NFAT (transcription factors involved in the replication of several viruses) [13]. Pentoxifylline augments leukocyte chemotoxicity and deformability, decreases leukocyte adhesion to the endothelium and natural killer cell activity, and promotes neutrophil degranulation, super-oxide release, monocyte-derived TNF-α production, IL-1-induced leukocyte responses, IL-6, TNF-α, and interferon-mediated responses, while suppressing T and B lymphocyte activation. The sum of these results suggests that this medicine may be useful in addressing the dysregulation of apoptosis and cytokine storms that characterize COVID-19 [14,15]. Consequently, pentoxifylline was validated for its anti-inflammatory and bronchodilatory effects, and it was found to be six times more effective than theophylline in terms of its anti-inflammatory effects [16].

There is evidence that COVID-19’s systemic involvements, particularly the lung, result in fibrotic pathogenic events. Pentoxifylline’s anti-TNF-α activity can explain its potential beneficial effects by boosting collagenase in fibroblasts and lowering formation of collagen, glycosaminoglycan, and fibronectin [17].

**Figure 1:**
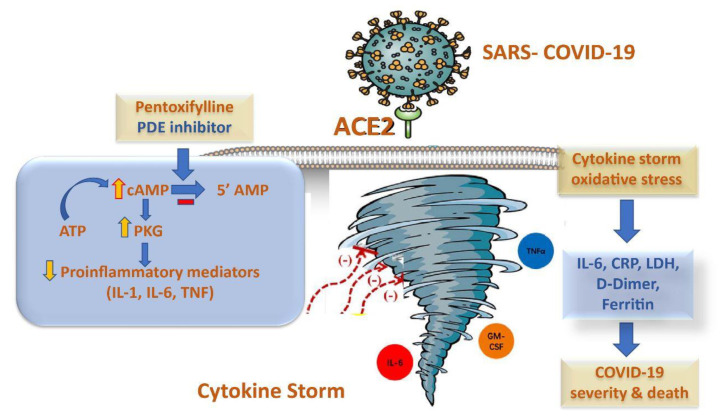
The potential anti-inflammatory mechanism of pentoxifylline in cytokine storm syndrome in COVID-19 patients.

Another mechanism of action for PTX has been recently proposed by researchers. As shown in Figure 1, adenosine A2A receptors (A2ARs) are seven-pass G-protein-coupled receptors that increase adenylate cyclase activity This, in turn, leads to an increase in the production of intracellular cAMP in multiple cells, including neutrophils, T-cells, macrophages, endothelial cells, natural killer cells, and platelets. These stages of development may prove useful in dealing with SARS-CoV-2 infections and their subsequent effects [18,19]. 

Looking at organ failure, particularly respiratory failure, which is the final fatal manifestation of COVID-19, the protective effect of pentoxifylline in organ injuries may have a great benefit in those patients [20]. It has been demonstrated that pentoxifylline is an effective treatment for ARDS as well as for respiratory failure [21,22]. Several studies have shown that pentoxifylline has the potential to lessen the impact on vital organs such as the heart, liver, kidneys, and brain [23,24,25,26]. Pentoxifylline helps to control sepsis more efficiently and reduces the mortality rate associated with it in both adults and neonates [27,28]. According with these findings, numerous researchers have endorsed pentoxifylline as an effective COVID-19 therapy [20,29,30].

An external pilot study carried on moderate and severe cases of COVID-19-infected patients studied pentoxifylline’s effect on many parameters. This study demonstrated an increase in the level of lymphocyte count and decrease in serum LDH, while recording no statistically significant difference in the number of days patients were hospitalized, the number of deaths, or the percentage of patients who required intubation, but all three trends were noted. These findings suggest that pentoxifylline may be reclassified as a medicine for COVID-19 treatment due to its great safety profile, accessibility, and lack of danger of immunosuppression; nevertheless, these data must be validated in pragmatic randomized controlled studies [31]. 

In brief, medications that inhibit viral replication and modulate the immune system can prevent viral infections. Pentoxifylline’s antiviral and anti-inflammatory actions on SARS-CoV-2 warrant further studies [32].

## 2. Results

### 2.1. Baseline Clinical Characteristics

**Figure 2:**
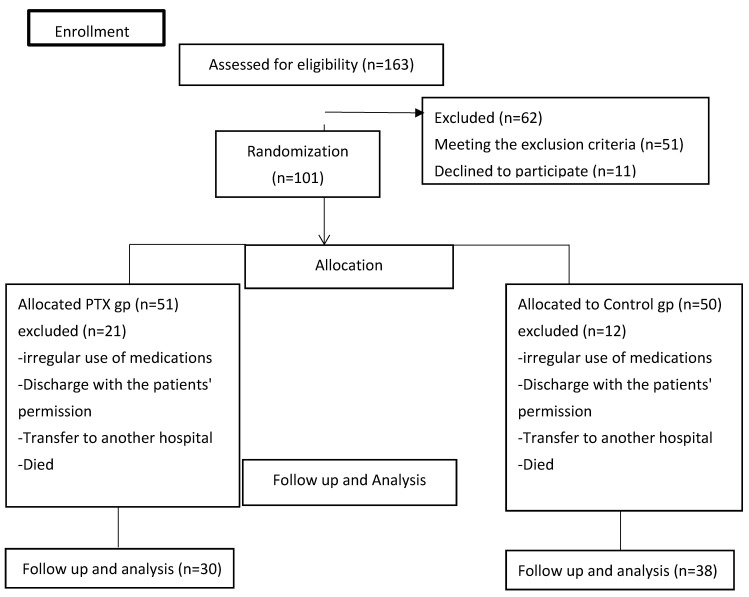
A CONSORT diagram of the study.

Out of a total of 163 COVID-19-infected hospital admissions, 101 patients matched the inclusion criteria. There was a total of 68 patients included (24 females, 44 males), and their data were divided into two groups (treatment and control) for the purpose of conducting an intention-to-treat analysis. A CONSORT diagram is shown in Figure 2. There was no significant difference between treatment and control groups in gender and age distribution, history of patient, or need for oxygen therapy as indicated in Table 1. 

### 2.2. Comparison between Initial and Outcome Treatment Laboratory Parameters

Initial laboratory values were compared between the two groups as shown in Table 2. There was no statistically significant difference between the two groups in any parameter. 

Initial laboratory values were compared to outcome laboratory values in both treatment and control groups as shown in Table 3. There was an improvement in C-reactive protein in both groups. However, the D-dimer level in the treatment group showed a significant increase while showing no statistical significant difference in the control group. There was a statistically significant difference in IL6, total leukocyte count, and neutrophil-to-lymphocyte ratio levels in both groups.

### 2.3. Comparison of Clinical Outcomes and Kaplan–Meier Curve

As represented in Table 4, there was no statistical significant difference between treatment and control groups in terms of clinical improvement, which was defined as an improvement in hypoxia symptoms, such as increase in oxygen saturation, rise P/F ratio, decrease need for oxygen supplementation as well as initial improvement in inflammatory markers, the death percentages, and the length of hospital stay.

**Figure 3:**
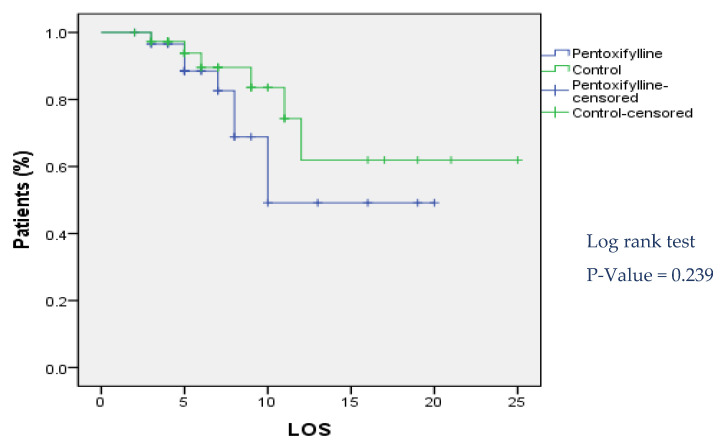
Kaplan–Meier curve for length of hospital stay.

There was no statistically significant difference between treatment and control groups in median length of stay days till discharge as represented in Figure 3.

### 2.4. Logistic Regression Model

A logistic regression was performed to predict the determinants of COVID-19 mortality by including covariates with *p*-value < 0.2 (ischemic heart diseases, asthma, IL-6, D-dimer and clinical improvement). The model explained 93.0% (Nagelkerke *R*2) of the variance in COVID-19 mortality and correctly classified 72.2% of cases. Patients with ischemic heart disease, asthma, and high initial IL-6 and D-dimer levels were associated with a significant increased likelihood of mortality but clinical improvement was associated with a significant reduction in the likelihood of COVID-19 mortality (Table 5).

Moreover, a logistics regression analysis to predict the determinants of clinical improvement outcome showed that a model including baseline LDH, IL-6, P/F ratio, length of stay, and hypertension predicted 38.3% of variation in clinical improvement (Nagelkerke *R*2) and correctly classified 74% of cases. Patients with higher P/F ratio were significantly associated with a significantly increased clinical improvement rate; however, patients with higher baseline IL-6 were associated with a significantly lower clinical improvement rate (Table 6).

## 3. Discussion

Patients with COVID-19 with cytokine storm syndrome might experience a wide variety of respiratory symptoms, including but not limited to pneumonia, dyspnea, rhinorrhea, upper airway congestion, cough, and pharyngalgia [33]. In the most severe cases, death can occur as a result of extensive damage to the alveoli and persistent respiratory failure [34]. The sudden onset of hypoxemic respiratory failure with bilateral infiltrates, sometimes known as acute respiratory distress syndrome (ARDS), is the primary characteristic of COVID-19 individuals who have severe disease [35]. Hyperinflammatory reaction, also known as cytokine storm, is often present in patients with severe cases of COVID-19 with ARDS [36]. C-reactive protein, ferritin, the neutrophil-to-lymphocyte ratio (NLR), and total leucocyte count (TLC), are some of the indicators monitored to track the inflammatory response and identify the clinical outcome [37].

Elevated levels of C-reactive protein have been identified in the serum of COVID-19 patients. In severe cases of COVID-19, an elevated CRP level is closely related to mortality [38]. Our results are in line with those that found in González-Pacheco et al.’s study [39] as after treatment with PTX, there was a decrease in CRP at *p* < 0.01 in both groups.

PTX contributed another potential advantage against COVID-19 severity, as seen in a reduction in IL-6, an important marker of cytokine storm syndrome, and C-reactive protein (CRP), according to a prospective trial by Chavarra et al. that involved 110 COVID-19 patients treated with normal therapy in combination with PTX and numerous antioxidants [40]; this agreed with our results as IL-6 decreased from 47.2 pg/mL. to 16.5 pg/mL. after treatment with PTX at *p* = 0.004.

In another randomized clinical trial conducted among hospitalized COVID-19 patients, 72/102 were analyzed. The intervention group included 400 mg PTX three times a day for ten days plus the standard regimen. The findings showed that PTX had no advantage over placebo in terms of improving hospital stay, clinical outcomes, or death rate. Further, PTX exhibited a positive effect on IL-6 and had an acceptable safety record [41] which was in line with our findings in decreasing IL-6 level and recording improvement in ALT levels in the study group after PTX treatment.

In the early stages of COVID-19, the ratio of neutrophils to lymphocytes (NLR) has been shown to be predictive of disease severity [42]. As a reliable sign of this infection, lymphocytopenia is useful in observing the development of COVID-19 pneumonia [43]. 

The neutrophil–lymphocyte ratio is high in the circulating blood of severely infected COVID-19 patients, indicating a decrease in lymphocytes and an increase in neutrophils [44].

Several investigations showed that severe cases of COVID-19 pneumonia that are related to cytokine storm were associated with lymphocytopenia (where lymphocyte count was below 1.5 × 10^9^/L) and neutrophilia (where neutrophil count was above 3–7.5 × 10^9^/L, the normal range) [45,46]. Furthermore, the lymphocyte counts of the both groups were present in higher levels after treatment.

Nevertheless, D-dimer levels increased significantly in the treatment group, whereas there was no statistically significant difference in the control group. This correlates with disease severity and death in hospitalized COVID-19 patients, similar to the findings of Yao et al. [47].

In a retrospective study, PTX were given to 58 out of 209 people. It was taken three times a day for seven days total. They showed a partial decrease in mortality and CRP levels, as well as an improvement in hypoxemia in the study group. There were no significant adverse reactions seen [37]. Additionally, a pilot study was conducted by Maldonado et al. among 38 moderate-to-severe COVID-19 patients. Similar to our findings, the data showed that PTX did not reduce the length of hospital stay or death rate significantly. They found that PTX significantly reduced LDH levels by 29.61% and raised lymphocyte counts by 64.25% without causing any adverse effects when compared to the control group [31]. 

Further, in patients suffering from a range of diseases, such as coronary artery disease, type 2 diabetes mellitus, idiopathic or ischemic cardiomyopathy, and chronic renal disease, a PTX treatment was found to have an anti-inflammatory impact, according to Brie et al.’s observations. The reduction in plasma concentrations of TNF-α and CRP provided additional evidence that the changes analyzed were statistically significant [14].

This study revealed that the mean number of clinical improvements showing in the length of stay of patients in the treatment group was 22 compared to 31 in the control group. In addition to the eight deaths in the therapy group, there were six in the control group. Furthermore, there is no statistical difference between the percentages of clinical improvement and mortality between the treatment and control groups. Pentoxifylline has a beneficial effect on certain inflammatory biomarkers as CRP and IL-6 and had no discernable adverse effects. There were some limitations in our study, summarized as the small sample size of treatment group and incomplete record data, including laboratory values and follow-up after a patient’s discharge. 

## 4. Patients and Methods

### 4.1. Study Design

This prospective open-label randomized controlled trial was conducted among hospitalized COVID-19 patients at the Teachers’ Hospital, Cairo, Egypt from November 2020 to April 2021. The study was approved by the Ethics Committee of the Faculty of Pharmacy at Beni-Suef University (REC-H-PhBSU-22007). Additionally, the study was registered at the clinical trials registry (ClinicalTrials.gov; NCT04739345). It was performed in accordance with the Declaration of Helsinki. Each participant was informed about the study’s aims and methodology before participation. The intervention was carried out only after they signed a consent form, protecting their confidentiality. The informed consent was obtained from a legally authorized representative (LAR) on behalf of the studied subjects. The informed consent was obtained after diagnosing the subjects with a cytokine storm, and it was signed in the hospital by the LAR of each subject.

### 4.2. Study Population

Patients included in the study were aged 18 and older and were admitted to the intensive care unit with COVID-19, confirmed by reverse transcription polymerase chain reaction (RT-PCR) as well as chest CT scan, associated with cytokine storm syndrome, defined as an increase in inflammatory indicators such as C-reactive protein (CRP) > 100 mg/L or ferritin > 600 ng/mL, lactate dehydrogenase (LDH) > 200 U/L, and interleukin-6 level (IL-6) > 10 pg/mL. There must also be at least one of the following circumstances: rapid breathing (>20 breaths per minute), low blood oxygen levels (92%), a low ratio of arterial oxygen partial pressure to inspired oxygen fraction (PaO_2_/FiO_2_) (350), or an increase in the number and size of patches in the lungs that indicate consolidation [48,49]. Women who were breastfeeding or pregnant, patients with a history of PTX use, patients with brain or eye bleeding, patients with renal (GFR 30 mL/min) or hepatic (Child–Pugh C) impairment, and patients with hypersensitivity to PTX were excluded.

### 4.3. Study Intervention of the Patients

Along with the standard treatment, patients in the intervention group were treated with 400 mg PTX tablets three times daily for 7 days in addition to the standard therapy. The control group received the standard therapy only for the same days as in the intervention group. At the time of the study, the standard protocol for the treatment of COVID-19 for all patients included the following antiviral regimen: 400 mg of hydroxychloroquine as a single dosage then 200 mg every 12 h, 200 mg of remdesivir as a first dose, followed by 100 mg once daily for five days, lopinavir/ritonavir (200/100) tablets every 12 h for five days, and tocilizumab was given intravenously at a dose of 400 mg daily for one dose. For each patient, regular supportive treatment, unfractionated heparin or low molecular weight heparin prophylaxis, antibiotics, corticosteroid therapy, and routine anticoagulant doses were all considered.

### 4.4. Outcomes’ Measurements

Demographic information such as age, gender, health complications, and medication history were recorded for all patients. The primary outcome was represented in the follow up of the laboratory tests’ changes during the study, including interleukin 6 (IL-6), C-reactive protein (CRP), lactate dehydrogenase (LDH), D dimer, ferritin, total leucocyte count (TLC) and the neutrophil-to-lymphocyte ratio (NLR). The clinical improvement of patients, length of stay in hospital, and number of deaths were evaluated as secondary outcomes. All patients were examined daily for any possible side effects during hospitalization. The side effects of PTX were assessed through the determination of alanine aminotransferase (ALT) and aspartate aminotransferase (AST) levels and reported as safety outcomes. In case of any reported adverse drug reaction (ADR) causing a risk to life, the intervention was terminated, and all essential treatment measures were considered.

### 4.5. Biochemical Analysis

First, 5 mL of peripheral venous blood in a plane vacutainer tube was withdrawn for the assessment of a complete blood count (CBC) (using Sysmex XT-1800i, Kobe, Japan), liver functions, alanine transaminase (ALT), and aspartate aminotransferase (AST), using a spectrophotometric assay on Advia chemistry 2400 XPT, Siemens Healthineers (Erlangen, Germany), automated quantitative C-reactive protein (CRP), and lactate dehydrogenase (LDH) (using Roche Diagnostics, Mannheim, Germany).

Following this, 5 mL of peripheral venous blood was obtained in a vacutainer tube containing citrate for the assessment of an automated quantitative D-dimer using the D-Dimer Reagent Specification commercially available from HEALES, Shenzhen Housing Technology, China.

The enzyme-linked immunosorbent assay (ELISA) is an effective method for measuring cytokines. The Human IL-6 ELISA kit tested serum IL-6 (Shanghai Sunred Biological Technology Co., Ltd., Shanghai, China). Serum ferritin and troponin were measured using the Human Ferritin ELISA kit and a Human Troponin ELISA kit (eBioscience, San Diego, CA, USA). The manufacturer’s ELISA steps were followed.

### 4.6. Statistical Analysis

#### 4.6.1. Sample Size Calculation

Assuming a two-sided tail hypothesis and an alpha level of 0.05, our power calculation showed that a sample size of 50 patients gave us 80% power to detect an effect size f2 of 0.25 [50,51].

#### 4.6.2. Descriptive and Inferential Statistics

Categorical data are shown as numbers and percentages, and comparisons were made using the chi-square test and the Fisher exact test, as needed. For data with a normally distributed distribution, continuous variables are reported in terms of mean and standard deviation and compared using an unpaired *t*-test. The Shapiro–Wilk test was used for normality assessment. For non-normally distributed continuous data, the median and range were used for data presentation while the Mann–Whitney *U* test was used for statics comparison. The Wilcoxon sign rank test was used to compare paired non-normally distributed quantitative data.

The contribution of biochemical and clinical predictors to each outcome of interest was determined by regression analysis. In a univariate analysis, we included clinical covariates and additional variables linked with the outcome at a *p*-value < 0.2. The final model contained clinical predictors with a significance level of *p*-value < 0.05. Logistic regression was used to determine predictors of binary outcomes, where results are expressed in terms of co-efficient of determination for the overall model while the odds ratio and confidence interval were used to express the significance of each predictor in the model. Survival analysis was performed using log rank test comparing medians, and a Kaplan–Meier curve was used to present survival over time.

All statistical tests were conducted using a two-sided approach, and results deemed statistically significant had a *p* < 0.05. Every single statistical analysis was carried out using version 26 of the Statistical Package for the Social Sciences (SPSS) (SPSS Inc., Chicago, IL, USA).

## 5. Conclusions

According to the findings of our study, there were no significant improvements in the clinical outcomes of COVID-19 with cytokine storm syndrome patients when comparing PTX to the control group. In spite of this, PTX was found to have a favorable impact on inflammatory biomarkers with no observable adverse effects.

## Figures and Tables

**Table 1:** Baseline characteristics comparison among treatment and control groups.

	Treatment Group (n = 30)	Control Group (n = 38)	*p* -Value
*Age and Gender*			
Male no. (%)	19 (63.3%)	25 (65.8%)	0.83
Age in yrs. mean ± SD	63.3 ± 11.75	65.4 ± 11.87	0.46
*Need For Oxygen Therapy*			
Low Flow Oxygen no. (%)	10 (33.3%)	17 (44.7%)	0.455
High Flow Oxygen no. (%)	18 (60%)	19 (50%)	0.468
Mechanical Ventilation no. (%)	2 (6.7%)	2 (5.3%)	1.00
*Patient History*			
Hypertension no. (%)	18 (60%)	20 (52.6%)	0.54
Diabetes Mellitus no. (%)	11 (36.7%)	16 (42.1%)	0.65
Ischemic Heart Disease no. (%)	5 (16.7%)	9 (23.7%)	0.48
Atrial Fibrillation no. (%)	2 (6.7%)	0 (0%)	0.19
Asthma no. (%)	1 (3.3%)	7 (18.4%)	0.07
COPD no. (%)	3 (10%)	0 (0%)	0.08
Chronic Kidney Disease no. (%)	1 (3.3%)	1 (2.6%)	1.00
Chronic Liver Disease no. (%)	0 (0%)	1 (2.6%)	1.00
Malignancy no. (%)	2 (6.6%)	4 (10.5%)	0.94
Thyroid disorders no. (%)	2 (6.6%)	1 (2.6%)	1.00
Stroke no. (%)	1 (3.3%)	0 (0%)	0.20
Obesity no. (%)	3 (10%)	5 (13.1%)	0.521
Smoker no. (%)	6 (20%)	9 (23.6%)	0.49
*Treatment Medications*			
Remdesivir no. (%)	25 (83.3%)	34 (89.5%)	0.49
Lopinavir/Ritonavir no. (%)	1 (3.3%)	2 (5.4%)	1.00
Hydroxychloroquine no. (%)	0 (0%)	2 (5.3%)	0.50
Tocilizumab no. (%)	17 (56.7%)	20 (52.6%)	0.74

**Table 2:** Initial laboratory values comparison between the two groups.

	Treatment Group (n = 30) Median Initial Level (Range)	Control Group (n = 38) Median Initial Level (Range)	*p* -Value
**D-Dimer**	0.5 (4.2)	0.6 (7.4)	0.47
**LDH**	385.4 (646)	385.4 (509)	0.69
**Ferritin**	605 (3777)	1009 (4120)	0.38
**CRP**	112.7 (335)	93 (309.5)	0.98
**IL6**	47.2 (355.5)	52.2 (232)	0.814
**Serum Creatinine**	1.3 (6.1)	1.4 (10.7)	0.47
**TLC**	7.6 (27.5)	8.1 (30.3)	0.91
**NLP**	8.6 (22.3)	8.8 (29.3)	0.57
**ALT**	50 (338)	40 (1019)	0.380
**AST**	41.5 (179)	47 (503)	0.302
**P/F**	171.5 (122)	170 (144)	0.282

**Table 3:** Initial and outcome laboratory values comparison between two groups.

	Median Initial Level (Range)	Median Outcome Level (Range)	*p*-Value
*C-Reactive Protein*			
Treatment	112.7 (335)	37.5 (601.5)	<0.01
Control	93 (309.5)	27.2 (217.8)	<0.01
*D-Dimer*			
Treatment	0.5 (4.2)	1.3 (6.9)	<0.01
Control	0.6 (7.4)	0.9 (38.4)	0.21
*LDH*			
Treatment	385.4 (646)	440.4 (59.1)	0.15
Control	385.4 (509)	440.4 (221.3)	0.13
*Ferritin*			
Treatment	605 (3777)	774.5 (2480)	0.45
Control	1009 (4120)	775 (2460)	0.16
*IL6*			
Treatment	47.2 (355.5)	16.5 (65)	0.004
Control	52.2 (232.1)	17.95 (77)	0.001
*Total Leukocytic Count*			
Treatment	7.6 (27.5)	12.5 (25.7)	0.003
Control	8.1 (30.3)	10.6 (19.5)	0.002
*Neutrophil to Lymphocyte Ratio*			
Treatment	8.6 (22.3)	12.3 (46.7)	0.002
Control	8.8 (29.3)	12.3 (93.5)	0.001
*P/F*			
Treatment	171.5 (122)	214.5 (188)	0.328
Control	170 (144)	223 (161)	0.022
*ALT*			
Treatment	50 (338)	42 (213)	0.848
Control	40 (1019)	51(1633)	0.007
*AST*			
Treatment	41.5 (179)	36 (142)	0.216
Control	47 (503)	40 (505)	0.806

**Table 4:** Comparison of outcomes between treatment and control groups.

Outcomes	Treatment Group (n = 30)	Control Group (n = 38)	*p*-Value
Clinical Improvement no. (%)	22 (73.3%)	31 (81.6%)	0.42
Length of Hospital Stay (LOS), Median (Range)	6.5 (4.25)	6.5 (6.25)	0.918
Death no. (%)	8 (26.7%)	6 (15.8%)	0.27
Occurrence of Adverse Effects no. (%)			
Nausea and Vomiting	6 (20%)	4 (10.5%)	0.497
Abdominal Discomfort	8 (26.6%)	9 (23.6%)	0.421
Anorexia	3 (10%)	1 (2.6%)	0.619
Headache	4 (13.3%)	7 (18.4%)	0.167

**Table 5:** Predictors of COVID-19 mortality by binary logistic regression.

Variable	Odds Ratio	Confidence Interval	Significance (*p*-Value)
IHD	3.46	2.1–4.5	0.036 *
Asthma	2.75	1.5–3.9	0.041 *
Baseline IL-6	2.06	1.3–4.2	0.01 *
Baseline D-dimer	1.3	1.1–2.04	0.048 *
Clinical improvement	7.4	3.7–8.3	0.008 *

IHD: ischemic heart disease, IL-6: Interleukin 6, * indicates significant difference at *p*-value < 0.05.

**Table 6:** Predictors of COVID-19 patients’ clinical improvement by binary logistic regression.

	Odds Ratio	Confidence Interval	Significance (*p*-Value)
Baseline LDH	0.88	0.97–1.02	0.19
Baseline IL-6	0.69	0.45–0.88	0.04 *
P/F ratio	1.81	1.1–2.4	0.036 *
Length of stay	1.23	0.8–1.3	0.058
Hypertension	1.12	0.12–10.6	0.13

LDH: lactate dehydrogenase, IL-6: interleukin 6, P/F ratio: ratio between arterial partial pressure of oxygen (PaO_2_) and fraction of inspired oxygen (FiO_2_), * indicates significant difference at *p*-value < 0.05.

## Data Availability

Data available within article.
